# Supplementary material for: Quantitative Profiling of Nanoscopic Protein Aggregates Reveals Specific Fingerprint of TDP‐43‐Positive Assemblies in Motor Neuron Disease
Source: Adv Sci (Weinh). 2025 Sep 23;12(44):e05484. doi: 10.1002/advs.202505484 (PMC12667529; doi:10.1002/advs.202505484)
Supplement: Supplementary file 1 — Supporting Information [file ADVS-12-e05484-s001.pdf]

SUPPLEMENTARY MATERIAL FOR

Quantitative Profiling of Nanoscopic Protein Aggregates Reveals Specific Fingerprint of TDP-43-positive Assemblies in Motor Neuron Disease

This file contains:

Supplementary Figure 1: Schematic of tissue processing paradigm.

Supplementary Figure 2: TDP-43 assemblies are found in both neurologically normal and disease-derived brain extracts.

Supplementary Figure 3: Aggregates containing TDP-43 are found in both neurologically normal and disease brain extracts.

Supplementary Figure 4: TDP-43-positive assemblies differ in size and shape between neurologically normal and disease-derived brain extracts.

Supplementary Figure 5: Signatures of MND found in nanoscopic TDP-43 assemblies.

Supplementary Figure 6: Schematic of mass spectrometry sample preparation paradigm.

Supplementary Figure 7: Proteomic analysis of affinity-captured aggregate fraction from embedded extracts.

Supplementary Figure 8: Particle counts for target proteins of interest in embedded extracts.

Supplementary Figure 9: Immunohistochemistry of pTDP-43 in MND_{TDP}.

Supplementary Figure 10: RT-qPCR measure of TDP-43 pathology supports SiMPull characterisation of MND_{SOD} donor samples.

Supplementary Figure 11: Immunohistochemistry confirms evidence of TDP-43 pathology in MND_{SOD} samples.

Supplementary Figure 12: Extracellular inflammasomes containing Apoptosis-associated Speck-like protein containing a CARD (ASC) were not altered in MND.

Supplementary Table 1: Qualitative summary of immunohistochemistry evidence of TDP-43 pathology.

Additional files:

Supplementary Table 2: Summary of quantitative proteomic data for MND_{TDP} and MND_{SOD} aggregate fraction.

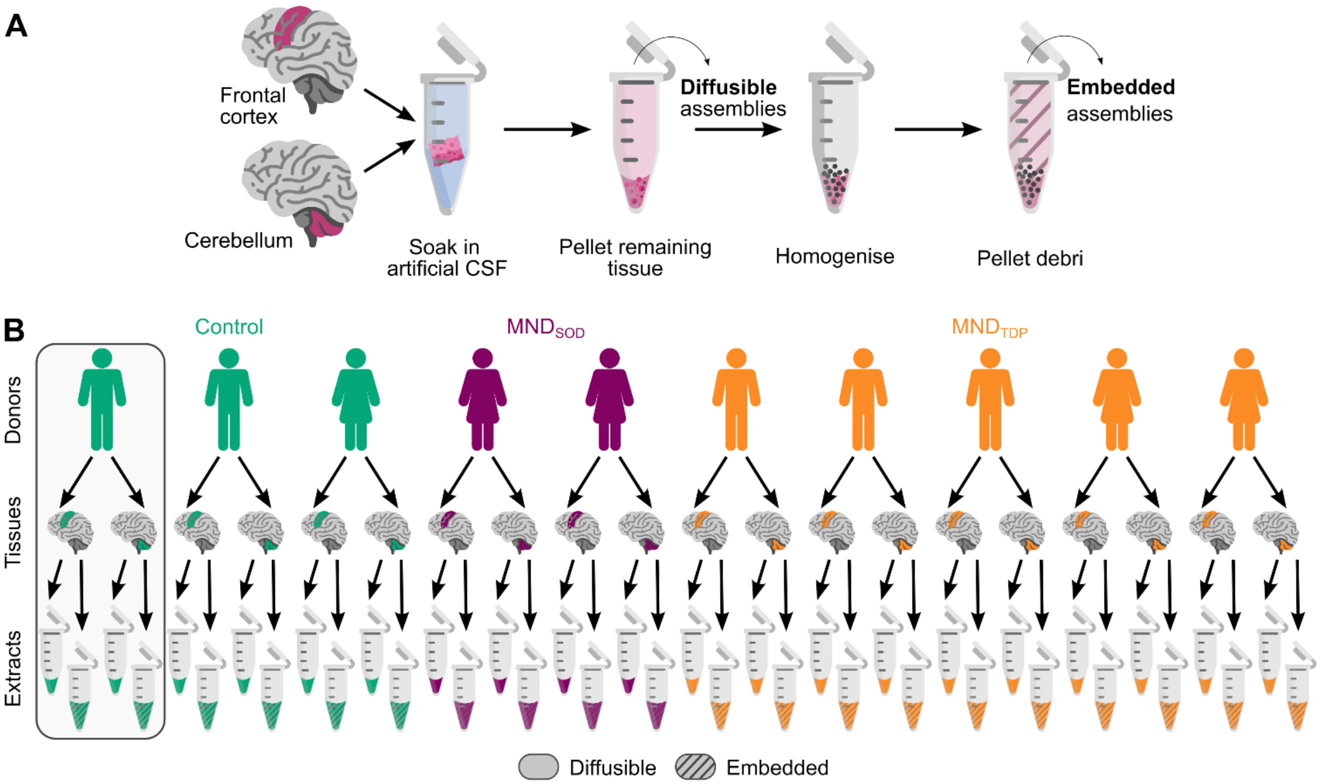

Supplementary Figure 1: Schematic of tissue processing paradigm. (A) Samples were first soaked for up to 2 h in artificial CSF buffer before the remaining intact tissue was pelleted via centrifugation and the supernatant was collected. The resultant supernatant was dialysed three times in 100× the sample volume, then finally collected as the "diffusible" extract. The remaining tissue was subjected to two rounds of bead-based homogenisation after which any cellular debris were pelleted via centrifugation and the resultant supernatant collected as the "embedded" extract. These extracts were then aliquoted and stored at -80 °C until use. **(B)** This processing pipeline was repeated for each tissue to generate a total of 40 extracts to be profiled. Matched frontal cortex and cerebellum tissues were obtained from a cohort of 10 donors, consisting of 5 with observed TDP-43 pathology at autopsy (MND$_{TDP}$) and 5 age-matched controls, including 2 disease-control donors diagnosed with SOD1-MND (MND$_{SOD}$) and 3 neurologically normal donors (CRL).

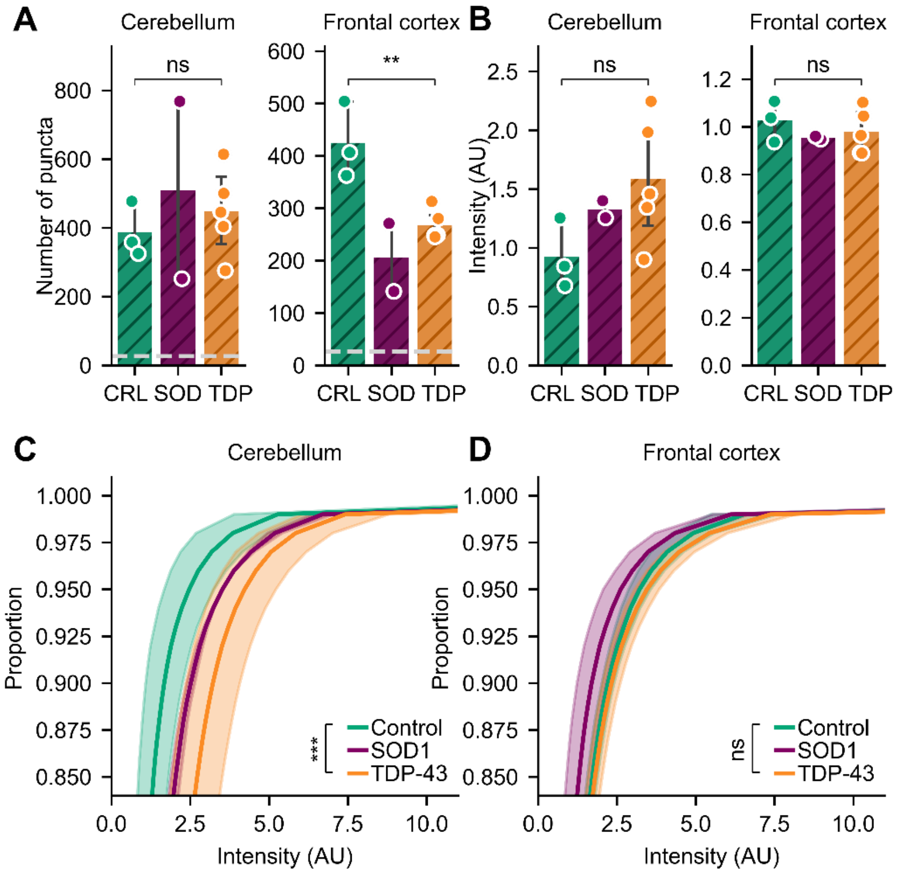

Supplementary Figure 2: TDP-43 assemblies are found in both neurologically normal and disease-derived brain extracts. (A) Number and **(B-D)** brightness of TDP-43 positive assemblies captured with flTDP-SiMPull from embedded extracts. Shown in *A*, *B* are the mean ± S.D. of donors in each category overlayed with the raw mean datapoints from three technical replicates of each donor, compared using Welch's t-test, ** $p < 0.01$, ns $p > 0.05$. Dashed line corresponds to an isotype control exposed to a reference sample of the same extract type. Shown in *C*, *D* are the mean interpolated cumulative distributions for each cohort ± S.D. of individual donors, compared via Kolmogorov–Smirnov test with Bonferroni correction, *** $p < 0.001$, ns $p > 0.05$.

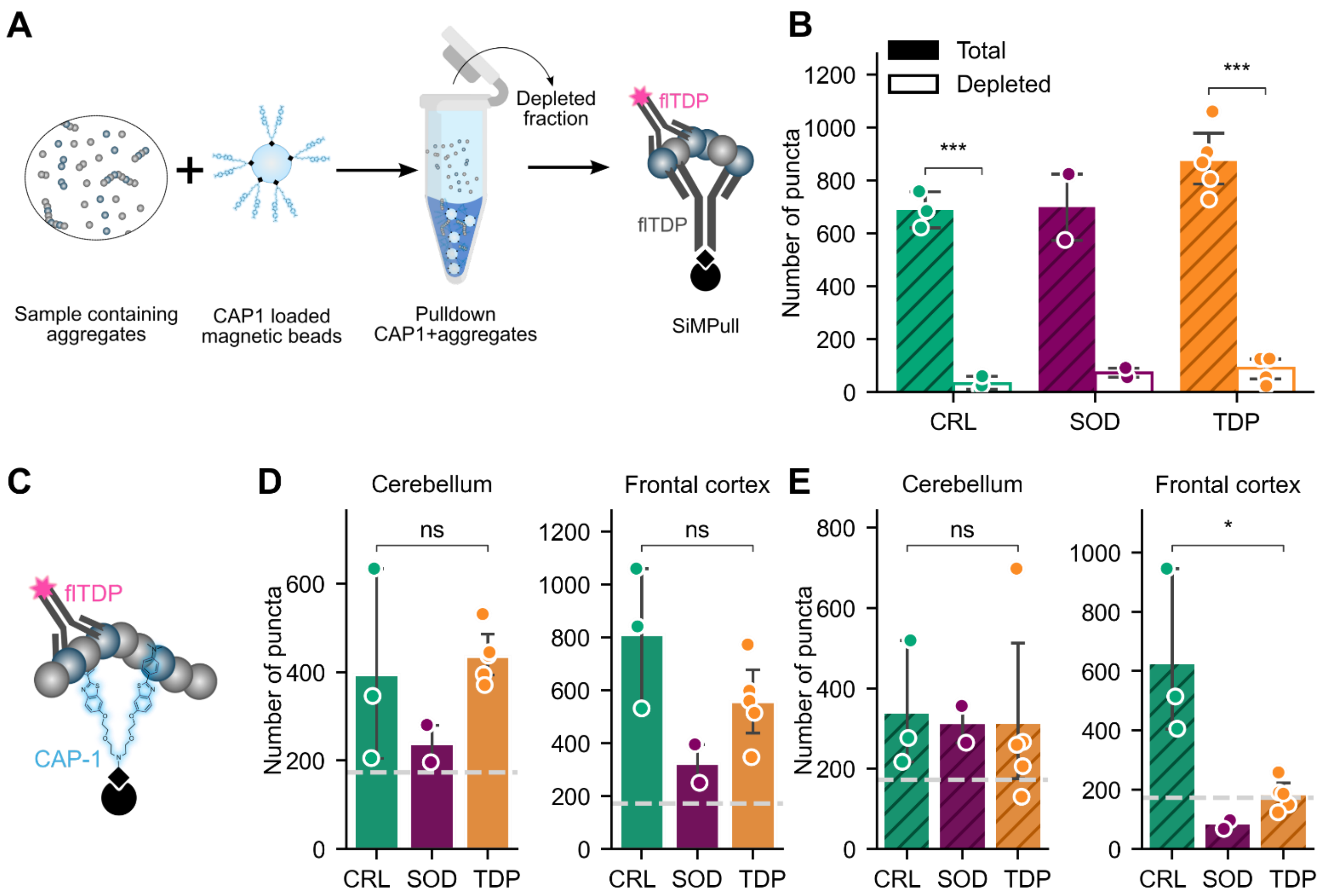

Supplementary Figure 3: Aggregates containing TDP-43 are found in both neurologically normal and disease brain extracts. (A) Schematic of CAP1-bead based aggregate affinity pulldown applied to embedded extracts followed by quantification via TDP-43-SiMPull. **(B)** Number of TDP-43 assemblies before (total) and after (depleted) CAP1 affinity capture of aggregates from the sample. Shown is mean ± S.D. of each cohort, overlayed with the raw mean datapoints from each donor, compared via two-way ANOVA ($p < 0.0001$) with post-hoc t-test with Holm-Bonferroni correction, *** $p < 0.001$. **(C)** Schematic of the CAP1-SiMPull setup applied to detect TDP-43 positive particles in *D, E*. **(D-E)** Number of TDP-43 positive particles in *(D)* diffusible and *(E)* embedded extracts captured with CAP1-SiMPull. Shown are the mean ± S.D. of each cohort, overlayed with the raw mean datapoints from three technical replicates of each donor. Dashed line corresponds to an isotype control exposed to a reference sample of the same extract type. Comparisons derived from Welch's t-test, * $p < 0.05$.

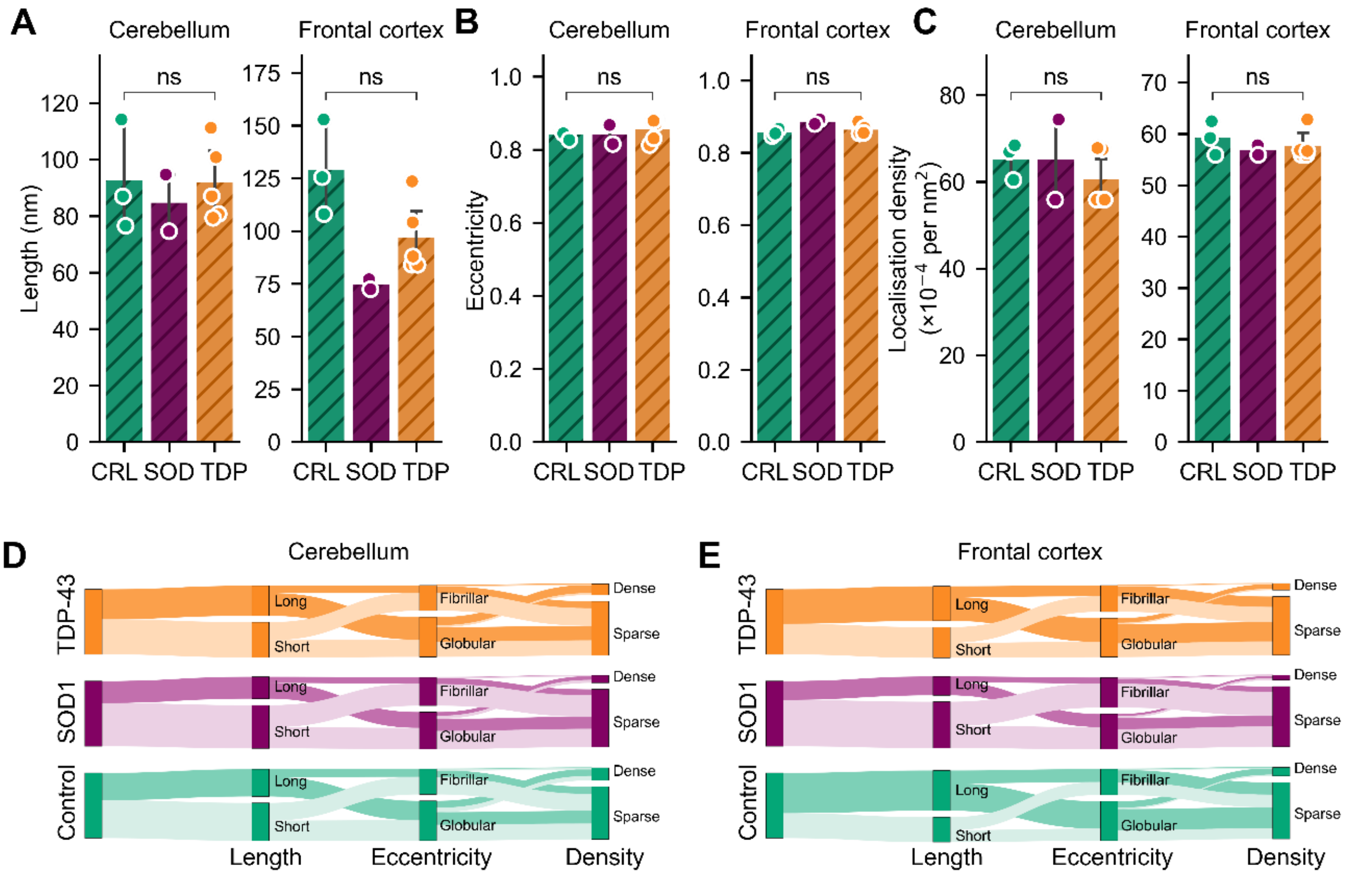

Supplementary Figure 4: TDP-43-positive assemblies differ in size and shape between neurologically normal and disease-derived brain extracts. (A-C) Average **(A)** length, **(B)** eccentricity, and **(C)** localisation density of TDP-43 positive assemblies captured with CAP1-SiMPull from embedded extracts. **(D-E)** Sankey visualisation of the proportion of assemblies that fall into each category as defined by cumulative thresholds for length (long > 100 nm), eccentricity (fibrillar > 0.9) and localisation density (dense > 0.001 localisations per nm^2). Shown in *A-C* are the mean ± S.D. of donors in each category overlayed with the raw mean datapoints from three technical replicates of each donor, compared using Welch's t-test. Shown in *D*, *E* are the compiled assembly populations for each cohort.

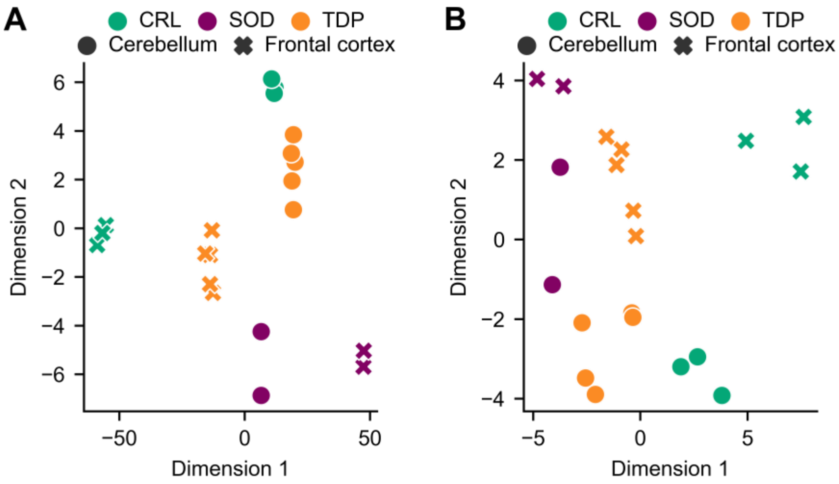

Supplementary Figure 5: Signatures of MND found in nanoscopic TDP-43 assemblies. Two-dimensional projection of linear discriminant analysis performed on (**A**) particle properties derived from embedded extracts or (**B**) combined particle properties from diffusible and embedded extracts characterised via SiMPull.

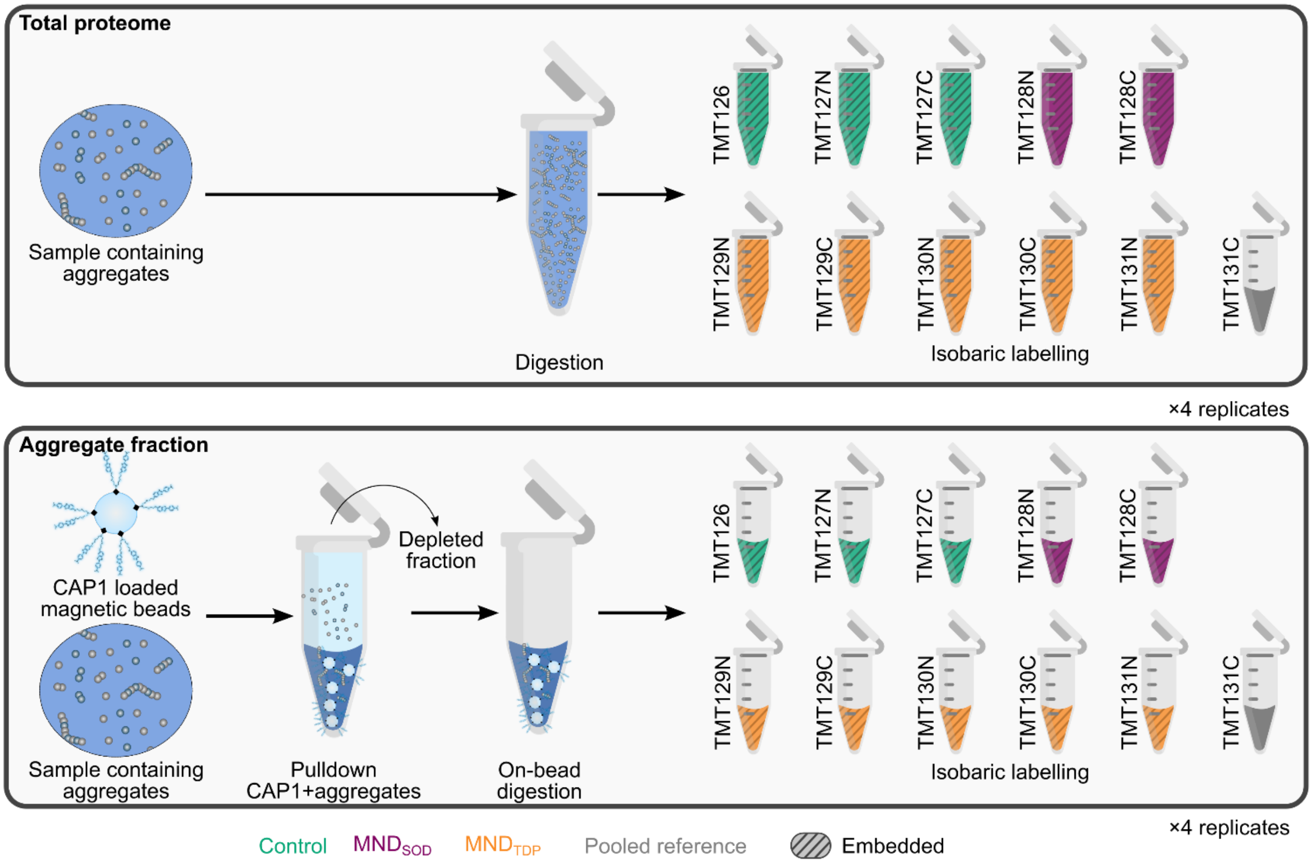

Supplementary Figure 6: Schematic of mass spectrometry sample preparation paradigm. Embedded extracts from the frontal cortex tissue were profiled using proteomics. Quadruplicate aliquots of each extract were taken as total proteome (upper panel) or subjected to CAP1-mediated pulldown of aggregates (aggregate fraction; lower panel) then subjected to labelling with one of 10 TMT labels (TMT 127N – TMT131N). Each set of 10 samples was then combined with a reference sample (labelled with the final TMT131C) to facilitate correction for batch effects, and the resultant pooled samples were then profiled by mass spectrometry.

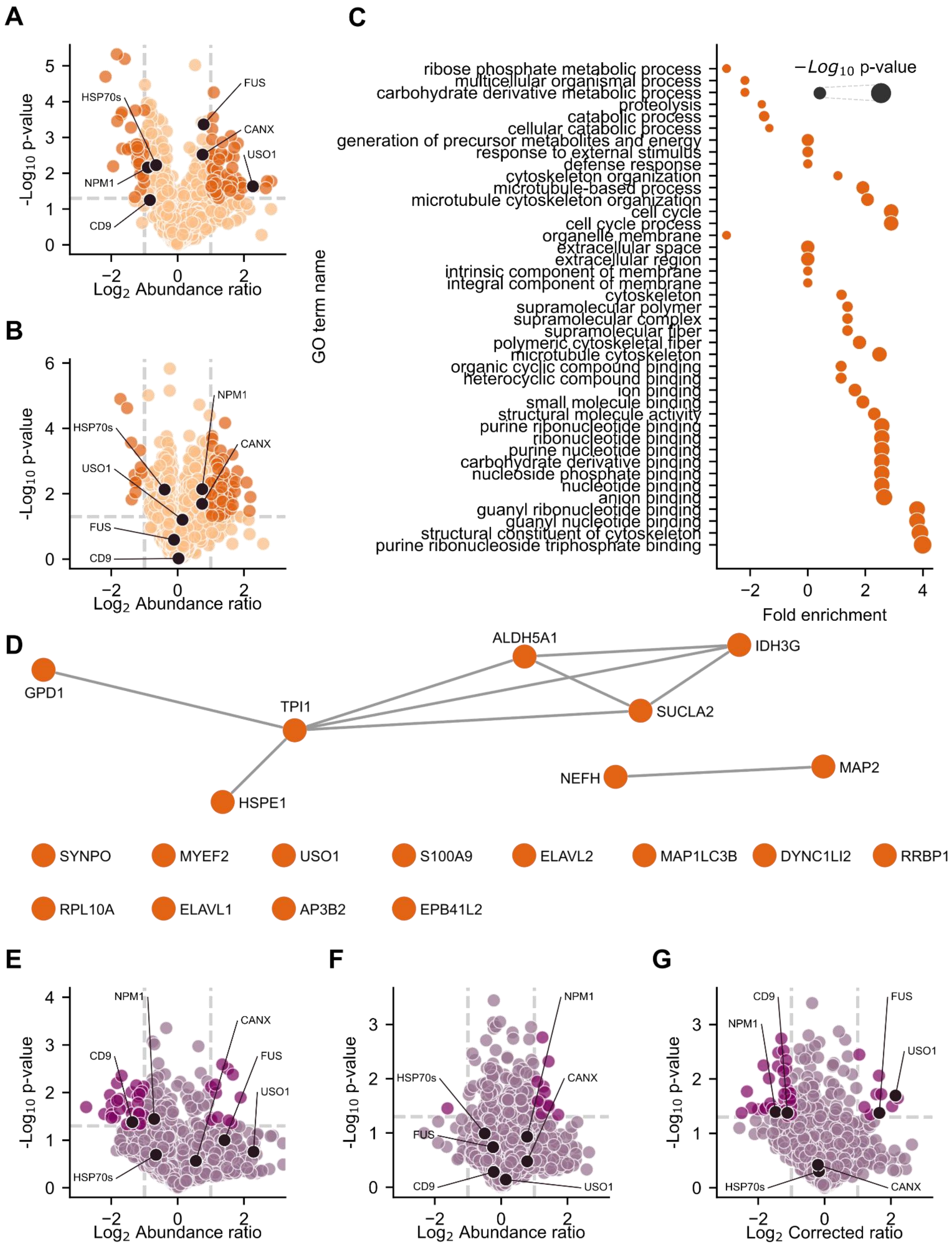
A
-Log10 p-value
Log2 Abundance ratio
HSP70s
NPM1
CD9
FUS
CANX
USO1
B
-Log10 p-value
Log2 Abundance ratio
HSP70s
USO1
FUS
CD9
NPM1
CANX
C
GO term name
Fold enrichment
−Log10 p-value
ribose phosphate metabolic process
multicellular organismal process
carbohydrate derivative metabolic process
proteolysis
catabolic process
cellular catabolic process
generation of precursor metabolites and energy
response to external stimulus
defense response
cytoskeleton organization
microtubule-based process
microtubule cytoskeleton organization
cell cycle
cell cycle process
organelle membrane
extracellular space
extracellular region
intrinsic component of membrane
integral component of membrane
cytoskeleton
supramolecular polymer
supramolecular complex
supramolecular fiber
polymeric cytoskeletal fiber
microtubule cytoskeleton
organic cyclic compound binding
heterocyclic compound binding
ion binding
small molecule binding
structural molecule activity
purine ribonucleotide binding
ribonucleotide binding
purine nucleotide binding
carbohydrate derivative binding
nucleoside phosphate binding
nucleotide binding
anion binding
guanyl ribonucleotide binding
guanyl nucleotide binding
structural constituent of cytoskeleton
purine ribonucleoside triphosphate binding
D
GPD1
TPI1
HSPE1
ALDH5A1
IDH3G
SUCLA2
NEFH
MAP2
SYNPO
MYEF2
USO1
S100A9
ELAVL2
MAP1LC3B
DYNC1LI2
RRBP1
RPL10A
ELAVL1
AP3B2
EPB41L2
E
-Log10 p-value
Log2 Abundance ratio
NPM1
CD9
HSP70s
CANX
FUS
USO1
F
-Log10 p-value
Log2 Abundance ratio
HSP70s
FUS
CD9
NPM1
CANX
USO1
G
-Log10 p-value
Log2 Corrected ratio
CD9
NPM1
HSP70s
FUS
USO1
CANX

Supplementary Figure 7: Proteomic analysis of affinity-captured aggregate fraction from embedded extracts. (A-B) Volcano plot depicting abundance ratio of proteins identified in **(A)** the CAP1 aggregate fraction and **(B)** the total proteome from frontal cortex embedded extracts of MND_{TDP} vs neurologically normal control cohorts. Thresholds depict $|log_2FC| > 1$ and $-log_{10}(p\text{-}value) > 1.3$ outside which proteins are considered significantly altered (dark orange). Proteins of interest (black) highlighted. **(C)** Panther GOSLIM gene ontology enrichment and **(D)** STRINGdb protein-protein interaction network for proteins enriched in the CAP1 aggregate fraction from MND_{TDP} embedded extracts. Nodes represent individual proteins connected. **(E-G)** Volcano plots depicting **(E)** abundance ratio of proteins identified in the CAP1 aggregate fraction, **(F)** abundance ratio of proteins identified in the total proteome, or **(G)** the corrected abundance ratio for proteins found in frontal cortex embedded extracts of MND_{SOD} vs neurologically normal control cohorts. Thresholds depict $|log_2FC| > 1$ and $-log_{10}(p\text{-}value) > 1.3$ outside which proteins are considered significantly altered (dark orange). Proteins of interest (black) highlighted.

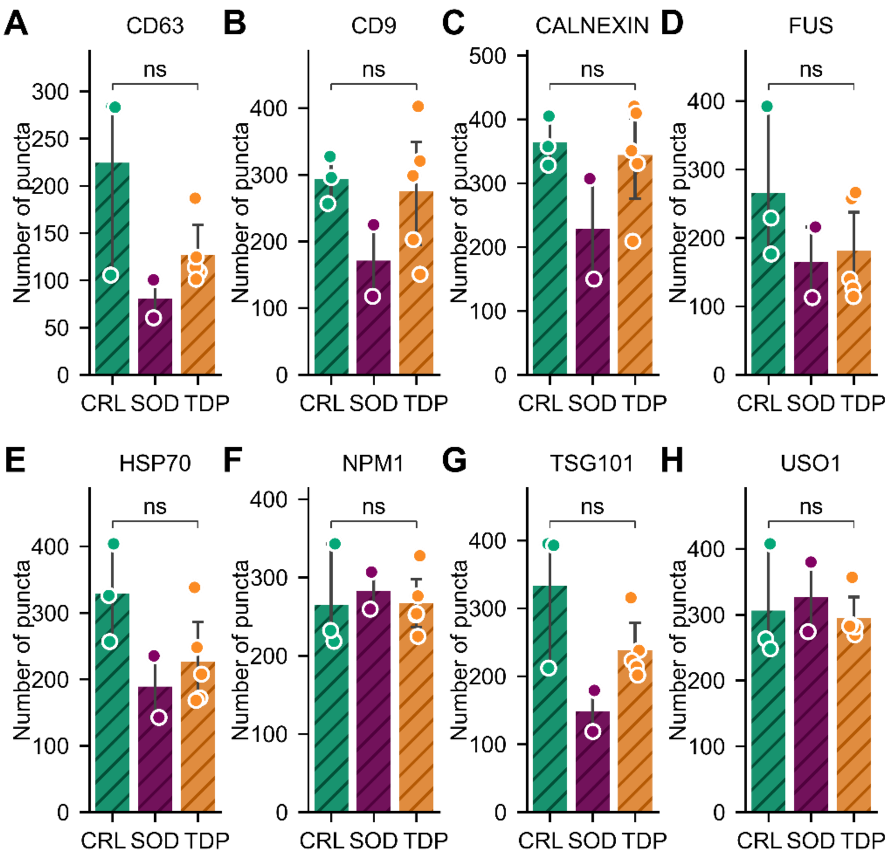

Supplementary Figure 8: Particle counts for target proteins of interest in embedded extracts. Number of aggregates captured with CAP1-SiMPull positive for **(A)** CD63, **(B)** CD9, **(C)** Calnexin, **(D)** FUS, **(E)** HSP70, **(F)** NPM1, **(G)** TSG101 and **(H)** USO1. Shown are the mean ± S.D. of donors in each category overlayed with the raw mean datapoints from three technical replicates of each donor compared using Welch's t-test, ns $p > 0.05$.

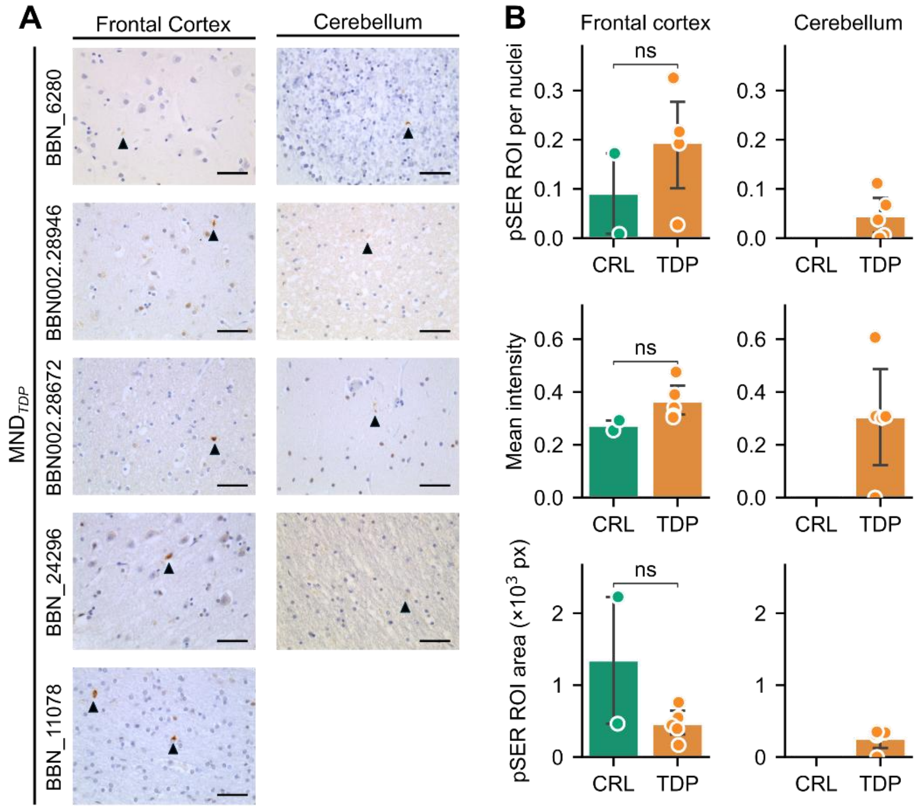

Supplementary Figure 9: Immunohistochemistry of pSER TDP-43 in MND$_{TDP}$. Immunohistochemical staining performed by the receiving brain bank using an antibody against phosphorylated TDP-43, used to categorise MND donor tissues as TDP-43-positive. **(A)** Exemplar images of MND$_{TDP}$ frontal cortex and cerebellum tissues. Black arrows indicate TDP-43 pathology detected by computational image analysis. Scale bars represent 50 µm. **(B)** pSER TDP-43 regions of interest (ROI) were quantified as DAB (3,3'-diaminobenzidine) puncta. DAB (pSER) and hematoxylin (nuclei) channels were separated computationally using scikit-learn, then regions of interest identified in both channels with area > 100 pixels and mean intensity > 0.25 AU. Shown are the mean ± S.D. of donors in each cohort for which slides were examined on intake, overlayed with the raw mean datapoints from ten fields of view collected for each available donor tissue. Statistical comparisons were completed using Welch's t-test, ns $p > 0.05$.

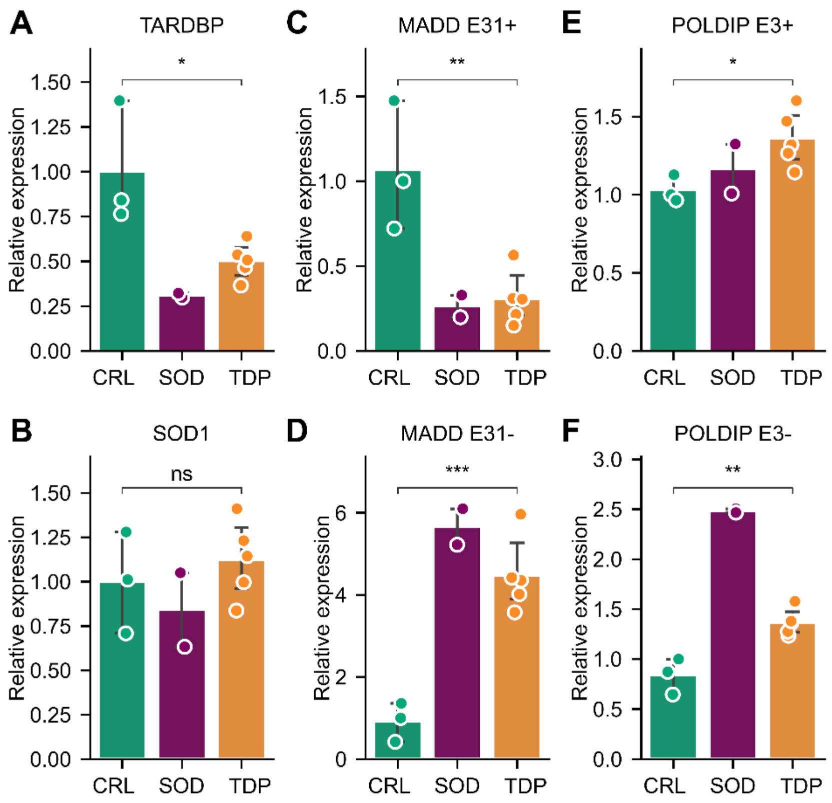

Supplementary Figure 10: RT-qPCR measure of TDP-43 pathology supports SiMPull characterisation of MND*SOD* donor samples. (A-F) Relative expression of six target genes in frontal cortex samples as measured by quantitative PCR (qPCR). Gene counts were normalised to reference genes *GAPDH*, *ACTB* and *YWHAZ.* Expression of canonical **(A)** TDP-43 and **(B)** SOD1 is shown, alongside two TDP-43 splicing targets **(C-D)** *MADD* and **(E-F)** *POLDIP* for which inclusion (+) of specific exons (E31 and E3 respectively) yields the canonical transcript, while exclusion (-) is associated with loss of TDP-43 function. Shown are the mean ± S.D. of donors in each category overlayed with the raw mean datapoints from three technical replicates of each donor compared using Welch's t-test, ns $p > 0.05$ * $p < 0.05$ ** $p < 0.01$ *** $p < 0.001$.

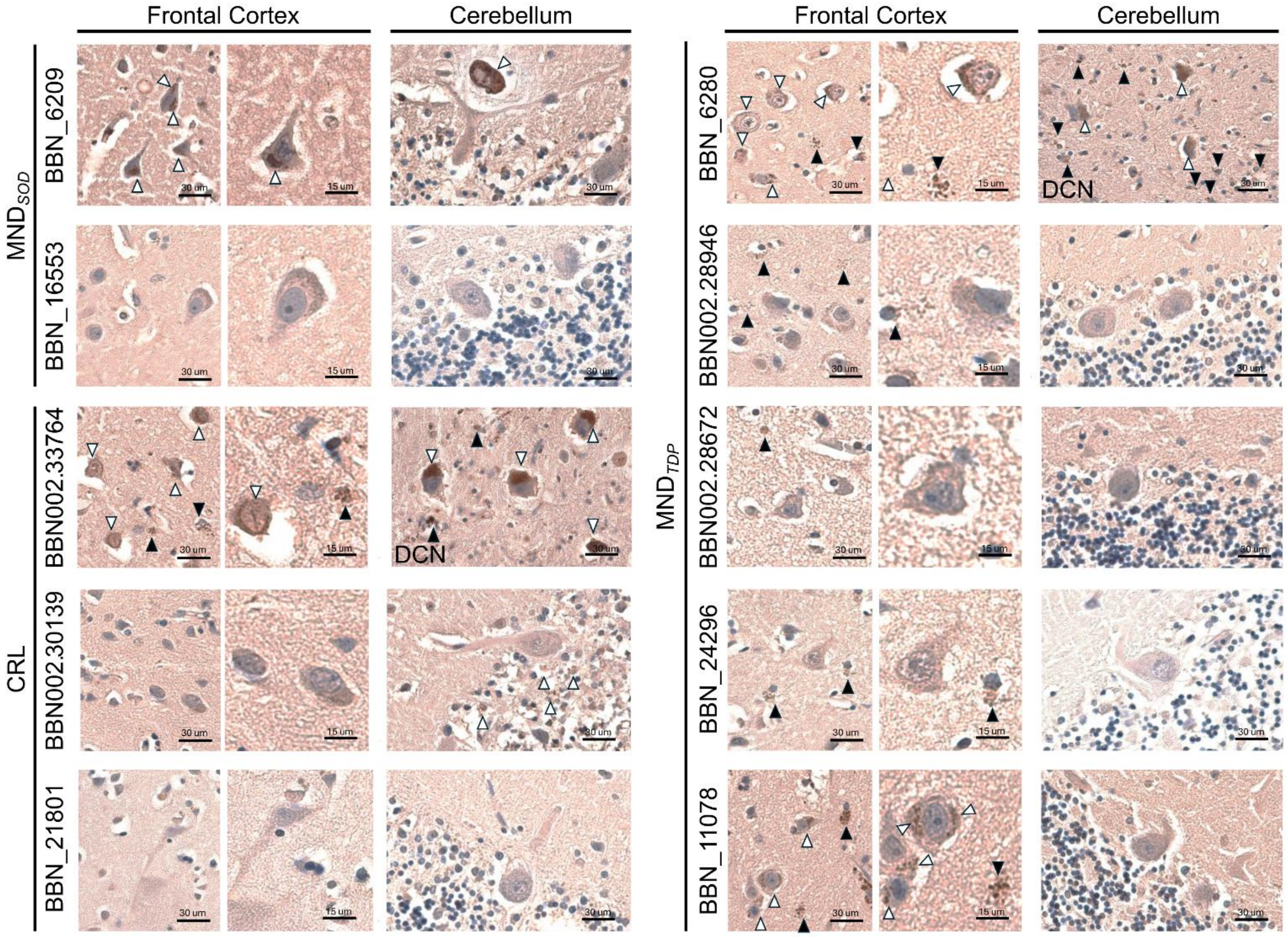

Supplementary Figure 11: Immunohistochemistry confirms evidence of TDP-43 pathology in MND$_{SOD}$ samples. Immunohistochemical staining performed using TDP-43 aptamer, used to detect, with high sensitivity, TDP-43 aggregates in both neurons and glia. Shown are exemplar images for each donor stained with TDP-43$_{APT}$[51,52] from frontal cortex and cerebellar sections. White arrows indicate neuronal TDP-43 pathology, black arrows indicate glial TDP-43 pathology, DCN indicates neurons within the deep cerebellar nuclei. See Supplementary Table 1 for summarised qualitative findings of TDP-43 pathology.

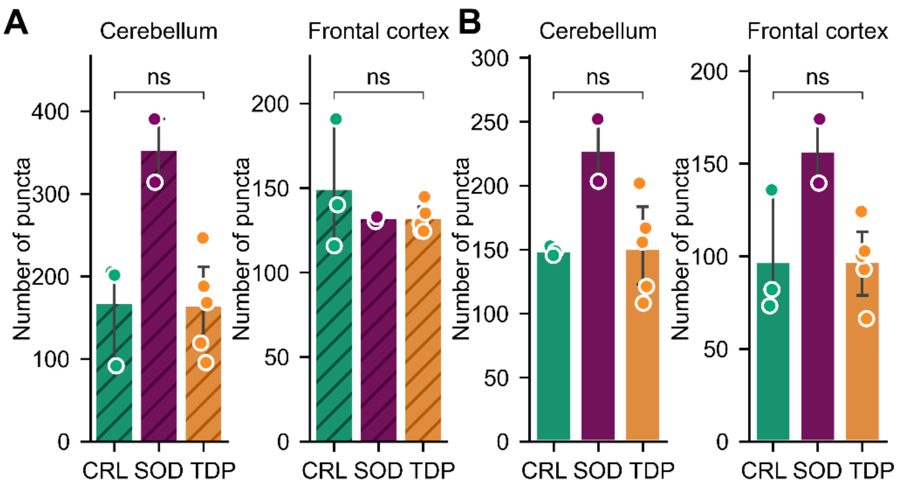

Supplementary Figure 12: Extracellular inflammasomes containing Apoptosis-associated Speck-like protein containing a CARD (ASC) were not altered in MND. (A-B) Number of ASC positive assemblies[39] captured from **(A)** embedded and **(B)** diffusible extracts. Shown are the mean ± S.D. of donors in each category overlayed with the raw mean datapoints from three technical replicates of each donor compared using Welch's t-test, ns $p > 0.05$.

Supplementary Table 1: Qualitative summary of immunohistochemical staining with the TDP-43 aggregate aptamer by qualified pathologist. See Supplementary Figure 9 for exemplar images highlighting TDP-43 pathology.

MRC BNN-ID	Category	TDP-43 pathology (TDP-43$_{APT}$)	
		Frontal Cortex	Cerebellum
BNN_6209	SOD1-MND	Abundant cytoplasmic TDP-43 pathology in neurons. No apparent glial pathology.	Heterotopic Purkinje cell containing abundant cytoplasmic TDP-43 pathology.
BBN_16553	SOD1-MND	No pathology.	No pathology.
BNN002.28672	TDP43-MND	Occasional glial TDP-43 pathology, no neuronal TDP-43 pathology.	No pathology.
BNN_24296	TDP43-MND	Abundant glial TDP-43 pathology, no neuronal TDP-43 pathology.	No pathology.
BNN_11078	TDP43-MND	Abundant neuronal and glial TDP-43 pathology.	No pathology.
BNN_6280	TDP43-MND	Abundant neuronal and glial TDP-43 pathology.	No TDP-43 pathology in grey matter but extensive TDP-43 pathology in deeper structures of the cerebellum (i.e. in cerebellar nuclei).
BNN002.28946	TDP43-MND	Occasional glial TDP-43 pathology.	No pathology.
BNN002.33764	Control	Abundant neuronal and glial TDP-43 pathology including nuclear and cytoplasmic pathology.	No TDP-43 pathology in grey matter but extensive TDP-43 pathology in deeper structures of the cerebellum (i.e. in cerebellar nuclei).
BNN002.30139	Control	No pathology.	Occasional TDP-43 pathology noted in granular cell layer.
BNN_21801	Control	No pathology.	No pathology.
